# Source-device-independent heterodyne-based quantum random number generator at 17 Gbps

**DOI:** 10.1038/s41467-018-07585-0

**Published:** 2018-12-18

**Authors:** Marco Avesani, Davide G. Marangon, Giuseppe Vallone, Paolo Villoresi

**Affiliations:** 1 Dipartimento di Ingegneria dell’Informazione, Università degli Studi di Padova, Via Gradenigo 6B, 35131 Padova, Italy; 2 Istituto di Fotonica e Nanotecnologie—CNR, Via Trasea 7, 35131 Padova, Italy

## Abstract

Random numbers are commonly used in many different fields, ranging from simulations in fundamental science to security applications. In some critical cases, as Bell’s tests and cryptography, the random numbers are required to be both private and to be provided at an ultra-fast rate. However, practical generators are usually considered trusted, but their security can be compromised in case of imperfections or malicious external actions. In this work we introduce an efficient protocol which guarantees security and speed in the generation. We propose a source-device-independent protocol based on generic Positive Operator Valued Measurements and then we specialize the result to heterodyne measurements. Furthermore, we experimentally implemented the protocol, reaching a secure generation rate of 17.42 Gbit/s, without the need of an initial source of randomness. The security of the protocol has been proven for general attacks in the finite key scenario.

## Abstract (web summary)

For many applications, quantum random number generation should be fast and independent from assumptions on the apparatus. Here, the authors devise and implement an approach which assumes a trusted detector but not a trusted source, and allows random bit generations at ~17 Gbps using off-the-shelf components.

## Introduction

The possibility of generating random numbers by quantum processes is an invaluable resource in cryptography. Nowadays, common solutions based on pseudo or classical random number generators rely on deterministic processes, which are in principle predictable. On the contrary, quantum mechanics guarantees, from a theoretic point of view, that the outcome of the measurement is completely unpredictable. However, in a paranoid scenario (the usual framework of device-independent protocols), any imperfection in the physical realization of a quantum random number generator (QRNG) may leak information correlated with the generated numbers, the so-called side information^1^. Such classical or quantum correlations could be exploited by an eavesdropper to correctly guess the measurement outcomes.

The maximal amount of randomness that can be extracted in presence of such side information is given by the so-called quantum conditional min-entropy^2^. Several approaches have been proposed to lower bound it, depending on the number of assumptions required on the devices used in the generator. For “fully trusted” QRNGs^3–5^, the min-entropy can be evaluated because pure input states and well characterized measurement devices are assumed (see ref. ^6^ for more details). In contrast, device-independent (DI) protocols, by exploiting entanglement, do not need any assumption: the violation of a Bell inequality directly bounds the min-entropy, without the need of trusting the generated state and the used measurement devices. Fully trusted QRNG, including all the commercial ones, are easy to realize, but they require strong assumptions for their use in cryptography. On the contrary, DI protocols offer the highest level of security, but their realization is still too demanding for any practical use^7–11^.

Semi-device-independent (Semi-DI) protocols^12^, are a promising approach to enhance the security with respect to standard “fully trusted” QRNG, achieving fast generation rate, dramatically larger than DI QRNG. These require some weaker assumptions to bound the side information. Such assumptions can be related to the dimension of the underlying Hilbert space^13,14^, the measurement device^6,11,15–17^ or the source^18^, for example the mean photon number^19^ or the maximum overlap^20^ of the emitted states.

In this work, we introduce a QRNG belonging to the family of the Semi-DI generators. In particular, we will describe a novel source-device-independent (Source-DI) protocol by exploiting continuous variable (CV) observables of the electromagnetic (EM) field. In previously realized CV-QRNGs^15,21^, random numbers were generated by using a homodyne detector that measures a quadrature of the EM field. We propose and demonstrate a CV-QRNG based on heterodyne detection in the Source-DI framework: we will show how it is possible to obtain a lower bound on the eavesdropper quantum side information (i.e., the conditional min-entropy) and to achieve, to our knowledge, the fastest generation rate in the Semi-DI framework. The advantages of heterodyne measurement over homodyne are multiple: beside offering better tomography accuracy than homodyne^22,23^, heterodyne measurement offers an increased generation rate since it allows a “simultaneous measurement” of both quadratures. In addition, the experimental setup is simplified with respect to the protocol based on homodyne introduced in^15^, as there is no need of an active switch to measure the two quadratures. Finally, it is possible to derive a constant lower bound on the conditional quantum min-entropy that does not change during the experiment. Our Source-DI protocol assumes a trusted detector but it does not make any assumption on the source: an eavesdropper may fully control it, manipulating it in order to maximize her ability to predict the outcomes of the generator. Such approach is very effective in taking into account any imperfect state preparation. Although these are the typical assumptions that hold for QRNGs in the Semi-DI framework, this protocol features an important difference. Previous protocols counteract the eavesdropper via an active measurement strategy on the state, which implies the need for additional randomness to certify the numbers. Instead here the removal of the active basis switch has a deep impact on the type of protocol implemented: in this scheme no external initial randomness is required, making it a randomness generation protocol and not a randomness expansion protocol, unlike previous Semi-DI and DI realizations. Moreover, we will show the results of a practical realization of the protocol with a compact fiber optical setup that employs only standard telecom components. The electric signals coming from the detectors are digitalized in burst mode by an oscilloscope and further post-processed, achieving an equivalent generation rate of secure random numbers > 17 Gbit/s.

## Results

### A heterodyne QRNG

In standard CV-QRNGs, random numbers are obtained by measuring with an homodyne detector a quadrature observable of the EM fields, typically prepared in a vacuum state. CV-QRNGs are characterized by high generation rates owing to the use of fast photodiodes instead of (slow) single-photon detectors: continuous spectrum of the observables typically assures more than one bit of entropy per measurement and the use of photodiodes with high-bandwidth allow to sample the quadratures at GSample/s. In our QRNG, we implement a heterodyne detection scheme where two “noisy quadrature observables” are measured simultaneously^24,25^. More precisely, an heterodyne measurement corresponds to the following positive operator value measurement (POVM) $\left\{ {{\hat{\mathrm {\Pi}}}_\alpha } \right\}_{\alpha \in {\Bbb C}}$ where

1 $${\hat{\mathrm {\Pi}}}_\alpha = \frac{1}{\pi }\left| \alpha \right\rangle \left\langle \alpha \right|,$$

and |*α*〉 is the coherent state with complex amplitude *α*. If we define *ρ*_A_ as the density matrix of the EM field, the output of the heterodyne measurement is represented by the random variable *X*

2 $$X = \left\{ {q,p} \right\},\quad q = \Re {\mathrm{e}}\left( \alpha \right),p = \Im {\mathrm{m}}\left( \alpha \right),$$

distributed according to the following probability density function known as Husimi function:

3 $${\mathrm{Q}}_{\rho _{\mathrm{A}}}(\alpha ) = {\mathrm{Tr}}\left[ {{\hat{\mathrm {\Pi}}}_\alpha \rho _{\mathrm{A}}} \right] = \frac{1}{\pi }\left\langle \alpha \right|\rho _{\mathrm{A}}\left| \alpha \right\rangle .$$

In an ideal scenario where the QRNG user (Alice) can trust the source of random states, such scheme has the immediate advantage of doubling the generation rate with respect to an homodyne receiver. As the “raw” random numbers *X* are typically not uniformly distributed, it is essential to process them with a randomness extractor^26^. A randomness extractor compresses the input string of raw numbers, such that the shorter output string is composed by i.i.d. random bits.

In a real implementation, any heterodyne measurement is discretized. This means that the possible outcomes *X*_*δ*_ of the measure are discrete with a resolution given by *δ*_*q*_ and *δ*_*p*_ for the two “quadratures”. The discretized version of the POVM element ${\hat{\mathrm {\Pi}}}_\alpha$ is then given by ${\hat{\mathrm {\Pi}}}_{m,n}^\delta = {\int}_{m\delta _q}^{(m + 1)\delta _q} {\kern 1pt} {\mathrm {d}}q{\int}_{n\delta _p}^{(n + 1)\delta _p} {\kern 1pt} {\mathrm {d}}p{\kern 1pt} {\hat{\mathrm {\Pi}}}_{q + ip}$ and the possible outputs are distributed according to a discretized version of the Husimi function:

4 $${\mathrm{Q}}_{\rho _{\mathrm{A}}}^\delta (m,n) = {\mathrm{Tr}}\left[ {{\hat{\mathrm {\Pi}}}_{m,n}^\delta \rho _{\mathrm{A}}} \right] = {\int}_{m\delta _{\mathrm{q}}}^{(m + 1)\delta _{\mathrm{q}}} {\kern 1pt} {\mathrm{dq}}{\int}_{n\delta _{\mathrm{p}}}^{(n + 1)\delta _{\mathrm{p}}} {\mathrm{dpQ}}_{\rho _{\mathrm{A}}}({\mathit{q}} + {\mathit{ip}}){\kern 1pt} .$$

In a fully trusted QRNG, when the source is trusted and the input state is pure (such as for the vacuum) or the privacy of the generated numbers is not a concern, the number of random bits that can be extracted per sample is given by the so-called classical min-entropy of *X*_*δ*_

5 $$H_{{\mathrm{min}}}\left( {X_\delta } \right) = - {\mathrm{log}}_2\left[ {\mathop {{{\mathrm{max}}}}\limits_{m,n} {\mathrm{Q}}_{\rho _{\mathrm{A}}}^\delta \left( {{\mathit{m}},{\mathit{n}}} \right)} \right]{\kern 1pt} .$$

However, ultrafast generation is worthless for cryptographic applications if the numbers are not secure and private. If security is important, quantum side information must be also taken into account and the conditional quantum min-entropy $H_{{\mathrm{min}}}(X{\mathrm{|}}{\cal E})$^2,27–29^ must be evaluated. We recall that in the Source-DI framework, an eavesdropper may have full control of the source and then may have some prior information on the generated numbers. We will show that with a heterodyne scheme it is possible to generate unpredictable and secure numbers also when the source of quantum states is controlled by the eavesdropper.

### A secure POVM-based QRNG

In our Source-DI framework, Alice does not make any assumption on *ρ*_A_, such as its dimension or purity: the source may be even controlled by a malicious QRNG manufacturer, Eve. This framework is well suited to deal with imperfect sources of quantum states^6^. On the contrary, Alice carefully characterizes her local measurement apparatus and trusts it.

In this scenario, Eve is assumed to prepare the state *ρ*_A_ to be measured. In particular, Eve will prepare *ρ*_A_ in order to maximize her guessing probability *P*_guess_ of the outcomes of Alice heterodyne measurement. If the state *ρ*_A_ is not pure, it can be prepared by Eve as a incoherent superposition of states $\tau _\beta ^{\mathrm{A}}$ with probabilities *p*(*β*), such as $\rho _{\mathrm{A}} = {\int} {\kern 1pt} p(\beta )\tau _\beta ^{\mathrm{A}}{{d}}\beta$. As shown below, for quantum state *ρ*_A_ with positive Glauber–Sudarshan representation, Eve optimizes her strategy by using $\tau _\beta ^{\mathrm{A}}$ that are coherent states.

When Eve generates the state $\tau _\beta ^{\mathrm{A}}$, the best option for her is to bet on the heterodyne outcome with higher probability, namely ${\mathrm{max}}_{m,n}\,{\mathrm{Tr}}\left[ {{\hat{\mathrm {\Pi}}}_{m,n}^\delta \tau _\beta ^{\mathrm{A}}} \right]$. On average, Eve’s probability of guessing correctly the output of the heterodyne measurement can be written as $P_{{\mathrm{guess}}}\left( {X_\delta {\mathrm{|}}{\cal E}} \right)$ = ${\int} {\kern 1pt} p(\beta ){\mathrm{max}}_{m,n}{\mathrm{Tr}}\left[ {{\hat{\mathrm {\Pi}}}_{m,n}^\delta \tau _\beta ^{\mathrm{A}}} \right]d\beta$. Having full control of the source, given the state *ρ*_A_, Eve chooses the decomposition $\left\{ {p(\beta ),\tau _\beta ^{\mathrm{A}}} \right\}$ that maximizes *P*_guess_. We note that the states $\hat \tau _k$ are, in general, not orthogonal. In such scenario, quantum correlations between Alice and Eve are modeled by a shared a pure bipartite state *ρ*_AE_. The states *τ*_k_ are related to the optimal measurement that Eve should perform on *ρ*_AE_ in order to maximize her guessing probability.

According to the Leftover Hash Lemma^27,30^, the extractable randomness in the presence of side information is quantified by the quantum conditional min-entropy

6 $$H_{{\mathrm{min}}}\left( {X_\delta {\mathrm{|}}{\cal E}} \right) = - {\mathrm{log}}_2P_{{\mathrm{guess}}}\left( {X_\delta {\mathrm{|}}{\cal E}} \right),$$

where $P_{{\mathrm{guess}}}\left( {X_\delta {\mathrm{|}}{\cal E}} \right)$ is maximum probability of guessing *X*_*δ*_ conditioned on the quantum side information ${\cal E}$

7 $$P_{{\mathrm{guess}}}\left( {X_\delta {\mathrm{|}}{\cal E}} \right) = \mathop {{{\mathrm{max}}}}\limits_{\begin{array}{c}\left\{ {p(\beta ),\tau _\beta ^{\mathrm{A}}} \right\}\end{array}} {\int} {\kern 1pt} p(\beta )\mathop {{{\mathrm{max}}}}\limits_{m,n} {\mathrm{Tr}}\left[ {{\hat{\mathrm {\Pi}}}_{m,n}^\delta \tau _\beta ^{\mathrm{A}}} \right]d\beta .$$

The maximization in (7) is performed over all possible decomposition $\{ {p(\beta ),\tau _\beta ^{\mathrm{A}}}\}$ that satisfy $\rho _{\mathrm{A}} = {\int} {\kern 1pt} p(\beta )\tau _\beta ^{\mathrm{A}}d\beta$. The above considerations are valid not only for the heterodyne measurement, but are correct for any POVM measurement (also with Hilbert spaces of finite dimensions).

**Fig. 1:**
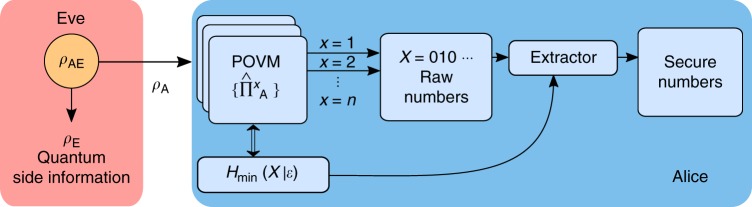
Structure of the Source-DI protocol. In the general Source-DI scenario, Eve prepares the state *ρ*_A_ that she sends to Alice such that her purification gives her the maximal guessing probability on Alice’s outcome. The structure of the POVM chosen by Alice to measure *ρ*_A_ already impose a lower bound on $H_{{\mathrm{min}}}(X{\mathrm{|}}{\cal E})$, independently from the input state or the output of her measurement (see Proposition 1). This bound is used to calibrate an extractor that returns, at each round of the protocol, secure random bits when applied to Alice’s outcome

Figure 1 represents a general protocol within this framework. In the case of infinite precision *δ*_*p*_*, δ*_*q*_ → 0 (i.e., the continuum limit) it is possible to define the differential quantum min-entropy as $h_{{\mathrm{min}}}\left( {X{\mathrm{|}}{\cal E}} \right)$ = ${\mathrm{lim}}_{\delta _p,\delta _q \to 0}[ {H_{{\mathrm{min}}}\left( {X_\delta {\mathrm{|}}{\cal E}} \right) + {\mathrm{log}}_2\delta _p\delta _q}]$^29^ and a corresponding $p_{{\mathrm{guess}}}(X{\mathrm{|}}{\cal E})$ = $2^{ - h_{{\mathrm{min}}}(X|{\cal E})}$. In this case *p*_guess_ is a probability density and not a proper probability such as *P*_guess_.

By exploiting the properties of POVMs, we derive a lower bound on $H_{{\mathrm{min}}}(X_\delta {\mathrm{|}}{\cal E})$ (and thus an upper bound on $P_{{\mathrm{guess}}}(X_\delta {\mathrm{|}}{\cal E})$).

#### Proposition 1

For any POVM, $\left\{ {{\hat{\mathrm {\Pi}}}_x} \right\}_{x \in X}$ the quantum conditional min-entropy $H_{{\mathrm{min}}}(X{\mathrm{|}}{\cal E})$ is lower-bounded by *H*_low_ = $- {\mathrm{max}}_{\left\{ {x \in X,\tau _{\mathrm{A}} \in {\cal H}_{\mathrm{A}}} \right\}}{\mathrm{log}}_2\left( {{\mathrm{Tr}}\left[ {{\hat{\mathrm {\Pi}}}_x\tau _{\mathrm{A}}} \right]} \right)$.

If the POVM reduce to projective measurements, the above bound is trivial, as it is always possible to find a state *τ*_A_ such that ${\mathrm{Tr}}\left[ {{\hat{\mathrm {\Pi}}}_x\tau _{\mathrm{A}}} \right] = 1$: in this case, no randomness can be extracted. However, for an overcomplete set of POVM we may have ${\mathrm{max}}_{\{ x,\tau _{\mathrm{A}}\} }{\mathrm{Tr}}\left[ {{\hat{\mathrm {\Pi}}}_x\tau _{\mathrm{A}}} \right] < 1$ and therefore randomness can always be extracted. We now exploit the above proposition for the specific case of heterodyne measurement.

#### Corollary 1

For the heterodyne measurement, the quantum conditional min-entropy is lower-bounded by

8 $$H_{{\mathrm{min}}}\left( {X_\delta {\mathrm{|}}{\cal E}} \right) \ge - {\mathop {{{\mathrm{max}}}}\limits_{\{ m,n,\tau _{\mathrm{A}}\} } {\mathrm{log}}_2\left( {{\mathrm{Tr}}\left[ {{\hat{\mathrm {\Pi}}}_{m,n}^\delta \tau _{\mathrm{A}}} \right]} \right)} = {\mathrm{log}}_2\frac{\pi }{{\delta _q\delta _p}}.$$

The corresponding differential min-entropy $h_{{\mathrm{min}}}\left( {X{\mathrm{|}}{\cal E}} \right)$ is lower-bounded by log_2_ *π*. The bounds are tight, i.e., $h_{{\mathrm{min}}}(X{\mathrm{|}}{\cal E}) = {\mathrm{log}}_2\pi$ and $H_{{\mathrm{min}}}\left( {X_\delta {\mathrm{|}}{\cal E}} \right)$ = ${\mathrm{log}}_2{\textstyle{\pi \over {\delta _q\delta _p}}} + O(\delta )$, for quantum state with positive Glauber–Sudarshan ${\cal P}(\alpha )$ representation.

The proofs of Proposition 1 and Corollary 1 are given in the Methods section. By using an heterodyne measurement scheme, a quantum tomography of the input state is also obtained^31^: although Alice generates the raw random numbers, she also reconstructs the state *ρ*_A_. Then it is possible to evaluate numerically the quantum conditional min-entropy by using (6) and (7). Although for a qubit system, this problem was elegantly addressed by^32^, it is not of easy solution in the CV case. On the other hand, Corollary 1 gives an easy lower bound on $H_{{\mathrm{min}}}\left( {X_\delta {\mathrm{|}}{\cal E}} \right)$. Alice knows that even if Eve forges a state with an optimal ${\cal E}$, such side information will not let Eve guess the heterodyne outcome with a probability larger than ${\textstyle{{\delta _q\delta _p} \over \pi }}$. In the presence on an imperfect source of quantum states, this is the most conservative strategy to adopt, but ensures the generation of completely secure random numbers while avoiding a complex numerical maximization (a discussion about the robustness of the protocol against general attacks can be found in the Methods section). It is worth to note that the min-entropy of the random numbers is bounded by a function that depends on the measurement resolution only. The measurement, in this scenario, is under control of the user: Alice can readily obtain the min-entropy (8) by measuring *δ*_*p*_ and *δ*_*q*_ of her well characterized apparatus. The min-entropy is constant and Alice does not need to worry updating its value, as long as she trusts the apparatus. In the case of imperfect heterodyne measurement Proposition 1 can be still used: the characterization of the measurement apparatus allows to define what are the actual POVM ${\tilde{\mathrm {\Pi}}}_{m,n}^\delta$ corresponding to such measurement. In Eq. (8) the ideal POVM ${\hat{\mathrm {\Pi}}}_{m,n}^\delta$ should replaced by the operators ${\tilde{\mathrm {\Pi}}}_{m,n}^\delta$. The bound ${\mathrm{log}}_2{\textstyle{\pi \over {\delta _q\delta _p}}}$ should be modified accordingly and its explicit value depends on the actual form of the operators ${\tilde{\mathrm {\Pi}}}_{m,n}^\delta$.

It is worth noticing that in many cases such lower bound is (almost) tight: indeed, coherent and thermal states have positive Glauber–Sudarshan ${\cal P}(\alpha )$ function and for those states the bound log_2_ *π* on the differential min-entropy is tight (the bound of the min-entropy is almost tight due to discretization). Moreover, in contrast to other Semi-DI QRNG where the min-entropy needs to be estimated in real time to provide security^13,15,20^, in our protocol it depends on the structure of the heterodyne POVM and it is always constant. Hence, Alice can apply on *X*_*δ*_ a randomness extractor calibrated on ${\mathrm{log}}_2 \hskip 2pt {\textstyle{\pi \over {\delta _q\delta _p}}}$ and erase any guessing advantage of Eve.

### Experimental implementation

**Fig. 2:**
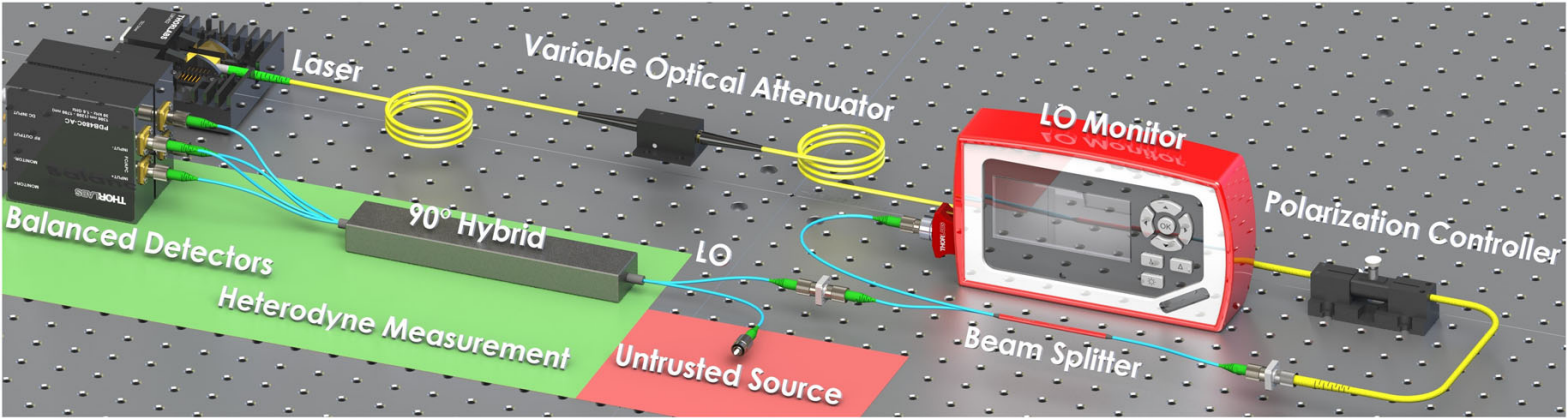
Schematic representation of the experimental setup. The setup consists of a 1550 nm laser used as a LO, measured in real time. The heterodyne detection is performed by a 90° optical hybrid and a pair of balanced InGaS detectors. The VOA is used during the calibration phase. Only commercial off-the-shelf devices were used

The proposed new protocol has been implemented with an all-fiber setup at telecom wavelength with the scheme in Fig. 2; in this way is possible to exploit the availability of fast off-the-shelf components for classical telecommunication while keeping the setup compact. The heart of the experiment lies in the heterodyne detection of the vacuum state, that samples the Q-function with the help of a coherent field |*α*〉 of a Local Oscillator (LO). As we work in the Source-DI scenario, from the point of view of security, the quantum state measured can be fully controlled by Eve, because we do not assume anything about the source. After the heterodyne detection, a 10-bit analog-to-digital converter (ADC) digitizes two analog signals, each one proportional to one of the quadrature (*q*, *p*).

**Fig. 3:**
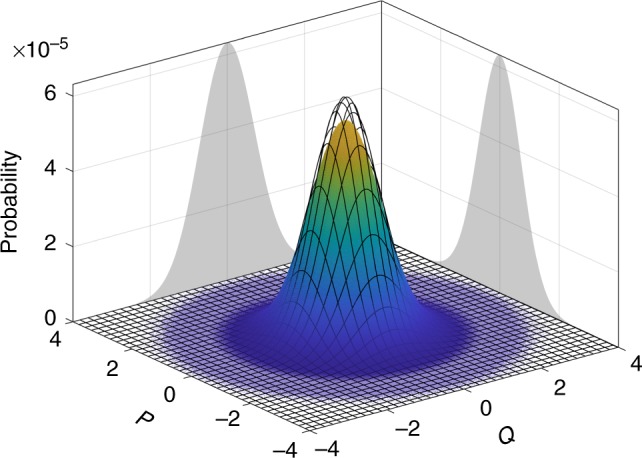
Experimental state tomography. The plot shows the Husimi function for the vacuum (meshed curve) and the measured state (colored histogram). The projections refer to the experimental data. The measured variance is slightly larger than the one expected for the vacuum due to the electronic noise that widens the distribution

These signals directly sample the Q-function in the phase space, as shown in Fig. 3. The resolution of the ADC can be directly converted to the equivalent resolution in the phase space, thanks to the calibration function (for more info see Supplementary Note 1); in our case we obtained *δq* = (14.05 ± 0.02) · 10^−3^ and *δp* = (14.14 ± 0.02) · 10^−3^, respectively.

The raw data are then digitally filtered, taking only a 1.25 GHz window in the central part of the spectrum obtained by the detectors. In such way the classical noise that is coupled with the detector is filtered. Finally, the data are downsampled at 1.25 GSample/s, matching the bandwidth of the signal and removing any correlation introduced by the oversampling.

We acquired 6 · 10^10^ measurements obtaining $\sigma _q^2$ = 0.55135 ± 0.00001 and $\sigma _p^2$ = 0.56732 ± 0.00001. As it can be seen from Fig. 3, the measured Q-function is slightly larger than the one expected for a pure vacuum state, where both variances are expected to be equal to 1/2. The increase of the variances is due to classical noise of the detectors: in our approach such noise is regarded as a “spreading” of the Q-function. Then, the effect of the electronic noise in reducing the generation rate is already included in our analysis for the quantum min-entropy. For more details see the Supplementary Notes 1 and 2.

The classical min-entropy *H*_min_(*X*_*δ*_) corresponds to the larger probability of output and it is given by

9 $$H_{{\mathrm{min}}}\left( {X_\delta } \right) = 14.100.$$

However, the quantum min-entropy can be lower-bounded by Eq. (8). With the quadrature resolutions used for the experiment, we obtain

10 $$H_{{\mathrm{min}}}(X_\delta {\mathrm{|}}{\cal E}) \ge 13.949,$$

for an equivalent secure generation rate of 17.42 Gbit/s. It is worth noticing that the high gain in security guaranteed by the conditional quantum min-entropy of Eq. (10) with respect to the classical min-entropy Eq. (9) implies a very small reduction of the generation rate (from 14.10 to 13.949 bits per sample). As said, such small reduction is experimentally owing to the electronic noise that slightly increases the quadrature variances with respect to the ideal value of 1/2. We also note that the generation rate can be improved by using an ADC with resolution larger than 10 bits.

In addition, these rates are not calculated in the asymptotic regime, i.e., in the limit of infinite repetitions of the protocol, but are valid for single-shot measurements. In fact, the conditional min-entropy $H_{{\mathrm{min}}}\left( {X_\delta {\mathrm{|}}{\cal E}} \right)$ is not estimated from the data, but it is bounded considering the structure of the POVM and the optimal strategy for the attacker, making it independent from the number of rounds of the protocol. Finally, a Toeplitz randomness extractor^33^ is calibrated using $H_{{\mathrm{min}}}\left( {X_\delta {\mathrm{|}}{\cal E}} \right)$, and extracts the certified numbers from the raw data. As a final check, we applied a series of statistical tests from the DieHarder and NIST suite: all of them are successfully passed (see Supplementary Note 4).

## Discussion

In this work, we demonstrated the versatility of heterodyne detection scheme for the generation of secure random numbers in a CV Source-DI framework, where no assumption on the source of quantum state is required. In fact, exploiting the properties of the POVM implemented by the heterodyne measurement, in Corollary 1 we obtained a direct lower bound to the conditional min-entropy, and hence on its security. This bound, also valid in the non-asymptotic regime, enables the user to erase all the side information related with an imperfect or malicious source of quantum states. Compared with previous Source-DI QRNGs^6,12,15^ this security is obtained without affecting the generation rate: in the previous protocols, part of the generated numbers were consumed to estimate and update the bound to the conditional min-entropy. In the protocol introduced here, the bound is constant, as it is determined by the resolution of the trusted measurement apparatus only. Hence, all the secure numbers are available to the user. Such simplification has many advantages for any practical implementation of the protocol. In particular, our protocol does not rely on external randomness to work, making it a standalone random number generator, whereas previous Semi-DI QRNG are based on randomness expansion protocols, that require either an initial seed or an external source of randomness to work.

Our approach allows to merge the speed of heterodyne measurements and the security of semi-DI protocols. Indeed, we realized the protocol with off-the-shelf components achieving, with an off-line post-processing, an equivalent rate of 17.42 Gbit/s,

## Methods

### Lower bound on the quantum conditional min-entropy

In this section, we give a proof of the proposition and the corollary that enable us to lower bound the quantum conditional min-entropy $H_{{\mathrm{min}}}\left( {X{\mathrm{|}}{\cal E}} \right)$.

*Proposition 1*. For any POVM, $\left\{ {{\hat{\mathrm {\Pi}}}_x} \right\}$ the quantum conditional min-entropy $H_{{\mathrm{min}}}(X{\mathrm{|}}{\cal E})$ is lower-bounded by $H_{{\mathrm{low}}}$ = $- {\mathrm{max}}_{\{ x,\tau _{\mathrm{A}} \in {\cal H}_{\mathrm{A}}\} }{\mathrm{log}}_2\left( {{\mathrm{Tr}}\left[ {{\hat{\mathrm {\Pi}}}_x\tau _{\mathrm{A}}} \right]} \right)$.

*Proof*. Given a set of POVM $\left\{ {{\hat{\mathrm {\Pi}}}_x} \right\}$, the maximum over *x* in (7) is bounded by ${\mathrm{max}}_x{\mathrm{Tr}}\left[ {{\hat{\mathrm {\Pi}}}_x\tau _\beta ^{\mathrm{A}}} \right]$ ≤ ${\mathrm{max}}_{x,\tau _{\mathrm{A}}}{\mathrm{Tr}}\left[ {{\hat{\mathrm {\Pi}}}_x\tau _{\mathrm{A}}} \right]$. Then Eq. (7) is upper bounded by:

11 $$\begin{array}{*{20}{l}} {P_{{\mathrm{guess}}}\left( {X{\mathrm{|}}{\cal E}} \right)_{{\mathrm{min}}}} \hfill & \le \hfill & {\mathop {{{\mathrm{max}}}}\limits_{\{ x,\tau _{\mathrm{A}}\} } {\mathrm{Tr}}\left[ {{\hat{\mathrm {\Pi}}}_x\tau _{\mathrm{A}}} \right]\mathop {{{\mathrm{max}}}}\limits_{\{ p(\beta ),\tau _B\} } {\int} {\kern 1pt} p(\beta )d\beta } \hfill \\ {} \hfill & = \hfill & {\mathop {{{\mathrm{max}}}}\limits_{\{ x,\tau _{\mathrm{A}} \in {\cal H}_{\mathrm{A}}\} } {\mathrm{Tr}}\left[ {{\hat{\mathrm {\Pi}}}_x\tau _{\mathrm{A}}} \right]} \hfill \end{array}$$

from which the bound on the min-entropy follows by using (6).

It is possible to specialize this result in the case of heterodyne detection, showing that the bound is always non-trivial:

*Corollary 1*. For the heterodyne measurement, the quantum conditional min-entropy $H_{{\mathrm{min}}}(X_\delta {\mathrm{|}}{\cal E})$ is lower-bounded by ${\mathrm{log}}_2{\textstyle{\pi \over {\delta _q\delta _p}}}$. The corresponding differential min-entropy $h_{{\mathrm{min}}}(X{\mathrm{|}}{\cal E})$ is lower-bounded by log_2_ *π*. The bounds are tight, i.e., $h_{{\mathrm{min}}}\left( {X{\mathrm{|}}{\cal E}} \right) = {\mathrm{log}}_2\pi$ and $H_{{\mathrm{min}}}\left( {X_\delta {\mathrm{|}}{\cal E}} \right)$ = ${\mathrm{log}}_2{\textstyle{\pi \over {\delta _q\delta _p}}} + O(\delta )$, for quantum state with positive Glauber–Sudarshan ${\cal P}(\alpha )$ representation.

*Proof*. It is well known that the Husimi function ${\mathrm{Q}}_{\rho _{\mathrm{A}}}({\mathit{q}} + {\mathit{ip}})$ is upper bounded by ${\textstyle{1 \over \pi }}$. Then, ∀*τ*_A_, the following inequality holds:

12 $$\begin{array}{c}{\mathrm{Tr}}\left[ {{\hat{\mathrm {\Pi}}}_{m,n}^\delta \tau _{\mathrm{A}}} \right] = {\int}_{m\delta _q}^{(m + 1)\delta _q} {\kern 1pt} dq{\int}_{n\delta _p}^{(n + 1)\delta _p} {\kern 1pt} dp{\mathrm{Q}}_{\rho _{\mathrm{A}}}\left( {{\mathit{q}} + {\mathit{ip}}} \right)\\ \le {\int}_{m\delta _{\mathrm{q}}}^{(m + 1)\delta _{\mathrm{q}}} {\kern 1pt} {\mathrm{dq}}{\int}_{n\delta _{\mathrm{p}}}^{(n + 1)\delta _{\mathrm{p}}} {\kern 1pt} {\mathrm{dp}}\frac{1}{\pi }\\ \le \frac{{\delta _{\mathrm{q}}\delta _{\mathrm{p}}}}{\pi }\end{array}$$

By Proposition 1, it follows that $H_{{\mathrm{min}}}(X_\delta {\mathrm{|}}{\cal E})$ ≥ ${\mathrm{log}}_2{\textstyle{\pi \over {\delta _q\delta _p}}}$. By the definition of differential quantum min-entropy as $h_{{\mathrm{min}}}(X{\mathrm{|}}{\cal E})$ = $\lim _{\delta _p,\delta _q \to 0}\left[ {H_{{\mathrm{min}}}\left( {X_\delta {\mathrm{|}}{\cal E}} \right) + {\mathrm{log}}_2\delta _p\delta _q} \right]$ it follows that $h_{{\mathrm{min}}}(X{\mathrm{|}}{\cal E}) \ge {\mathrm{log}}_2\pi$. To show the tightness, we note that any matrix *ρ*_A_ can be written as $\rho _{\mathrm{A}} = {\int} {\kern 1pt} {\cal P}(\alpha )\left| \alpha \right\rangle \left\langle \alpha \right|d^2\alpha$ where ${\cal P}(\alpha )$ is the Glauber–Sudarshan P-function. If ${\cal P}(\alpha )$ is positive it can be interpreted as a probability density and the state *ρ*_A_ can be seen as an incoherent superposition of coherent states. For small *δ*_*p*_ and *δ*_*q*_ the guessing probability of Eq. (7) becomes

13 $$P_{{\mathrm{guess}}}\left( {X_\delta {\mathrm{|}}{\cal E}} \right) = \delta _q\delta _p\mathop {{{\mathrm{max}}}}\limits_{\begin{array}{c}\{ p(\beta ),\tau _\beta ^{\mathrm{A}}\} \end{array}} {\int} {\kern 1pt} p(\beta )\mathop {{{\mathrm{max}}}}\limits_\alpha {\mathrm{Q}}_{\tau _\beta ^{\mathrm{A}}}(\alpha ) + O\left( {\delta ^3} \right).$$

As coherent states maximize the value of the Husimi function ${\mathrm{Q}}_{\tau _\beta ^{\mathrm{A}}}(\alpha )$, then the optimal decomposition in (13) is precisely $\left\{ {{\cal P}(\alpha ),\left| \alpha \right\rangle \left\langle \alpha \right|} \right\}$ such that $P_{{\mathrm{guess}}}(X_\delta {\mathrm{|}}{\cal E})$ = ${\textstyle{{\delta _q\delta _p} \over \pi }} + O(\delta ^3)$ and $H_{{\mathrm{min}}}\left( {X_\delta {\mathrm{|}}{\cal E}} \right)$ = ${\mathrm{log}}_2{\textstyle{\pi \over {\delta _q\delta _p}}} + O(\delta )$. The differential quantum conditional min-entropy is then exactly $h_{{\mathrm{min}}}(X{\mathrm{|}}{\cal E}) = \log _2\pi$.

### Security against coherent attacks

In the previous subsection we evaluated the quantum conditional min-entropy $H_{{\mathrm{min}}}^{(1)}(X{\mathrm{|}}{\cal E})$ for a single run of the protocol. Usually this corresponds to consider security against only individual attacks. However, as we calculate the min-entropy on the worst state *τ*^(1)^ that is allowed by physics, this result holds also for coherent attacks. In this section, we will show it explicitly, by bounding the min-entropy for *n* runs of the protocol $H_{{\mathrm{min}}}^{(n)}(X{\mathrm{|}}{\cal E})$ in terms of the min-entropy for a single run of the protocol $H_{{\mathrm{min}}}^{(1)}(X{\mathrm{|}}{\cal E})$. When Eve performs a coherent attack, she can prepare a general n-partite state $\hat \tau ^{(n)}$ to maximize her probability of guessing the *n* outcomes of Alice measurements, that can be written as

14 $${\hat{\mathrm {\Pi}}}_{\mathit{x}} \equiv {\hat{\mathrm {\Pi}}}_{x_1} \otimes {\hat{\mathrm {\Pi}}}_{x_2} \otimes \cdots \otimes {\hat{\mathrm {\Pi}}}_{x_n}.$$

The guessing probability of Eve for *n* runs of the protocol $P_{{\mathrm{guess}}}^{(n)}\left( {X{\mathrm{|}}{\cal E}} \right)$ can be written as

15 $$P_{{\mathrm{guess}}}^{(n)}(X{\mathrm{|}}{\cal E}) = \mathop {{{\mathrm{max}}}}\limits_{\{ x_i\} } \left[ {\mathop {{{\mathrm{max}}}}\limits_{\tau ^{(n)}} {\mathrm{Tr}}\left[ {\left( {{\hat{\mathrm {\Pi}}}_{x_1} \otimes \cdots \otimes {\hat{\mathrm {\Pi}}}_{x_n}} \right)\tau ^{(n)}} \right]} \right]$$

16 $$= \mathop {{{\mathrm{max}}}}\limits_{\{ x_i\} } \left[ {\mathop {{{\mathrm{max}}}}\limits_{\tau _1} {\mathrm{Tr}}\left[ {{\hat{\mathrm {\Pi}}}_{x_1}\hat \tau _1} \right] \cdots \mathop {{{\mathrm{max}}}}\limits_{\tau _n} {\mathrm{Tr}}\left[ {\hat {\Pi}_{x_n}\hat \tau _n} \right]} \right]$$

17 $$= \mathop {\prod}\limits_{i = 1}^n \left( {\mathop {{{\mathrm{max}}}}\limits_{x_i,\tau _i} {\mathrm{Tr}}\left[ {\hat {\Pi}_{x_i}\tau _i} \right]} \right)$$

18 $$= \left[ {P_{{\mathrm{guess}}}^{(1)}(X{\mathrm{|}}{\cal E})} \right]^n$$

where $P_{{\mathrm{guess}}}^{(1)}(X{\mathrm{|}}{\cal E})$ is the guessing probability for one run of the protocol, derived in the main text. In the above equations the state *τ*^(*n*)^ is a generic *n*-partite state, whereas *τ*_*i*_ are generic single-party states. The crucial step is going from Eqs. (15) and (16). The argument of the outer maximization in Eq. (15) is given by ${\mathrm{max}}_{\tau ^{(n)}}{\mathrm{Tr}}\left[ {{\hat{\mathrm {\Pi}}}_{\mathrm{x}}\tau ^{(n)}} \right]$ and corresponds to the maximum eigenvalue of the operator ${\hat{\mathrm {\Pi}}}_{\mathrm{x}}$. As ${\hat{\mathrm {\Pi}}}_{\mathrm{x}}$ is the product of Hermitian operators with non-negative eigenvalues, its maximum eigenvalue is equal to the product of their maximum eigenvalues, namely ${\mathrm{max}}_{\tau _1}{\mathrm{Tr}}\left[ {{\hat{\mathrm {\Pi}}}_{x_1}\hat \tau _1} \right] \cdots {\mathrm{max}}_{\tau _n}{\mathrm{Tr}}\left[ {{\hat{\mathrm {\Pi}}}_{x_n}\hat \tau _n} \right]$. This means that Eve’s optimal strategy is to generate a *n*-mode separable state *τ*^(*n*)^ = *τ*_1_ ⊗ *τ*_2_ ⊗ … ⊗ *τ*_*n*_.

Therefore, the min-entropy for *n* runs of the protocol $H_{{\mathrm{min}}}^{(n)}(X{\mathrm{|}}{\cal E})$ can be written as:

19 $$\begin{array}{*{20}{l}} {H_{{\mathrm{min}}}^{(n)}(X{\mathrm{|}}{\cal E})} \hfill & = \hfill & { - {\mathrm{log}}_2P_{{\mathrm{guess}}}^{(n)}(X{\mathrm{|}}{\cal E})} \hfill \\ {} \hfill & = \hfill & { - {\mathrm{log}}_2\left[ {\left( {P_{{\mathrm{guess}}}^{(1)}(X{\mathrm{|}}{\cal E})} \right)^n} \right]} \hfill \\ {} \hfill & = \hfill & {nH_{{\mathrm{min}}}^{(1)}(X{\mathrm{|}}{\cal E}).} \hfill \end{array}$$

Hence, our bound on the min-entropy is valid not only in the single-shot regime, but also for *n* repetitions of the protocol and coherent attacks.

### Experimental details

We employed a narrow linewidth ECL laser at 1550 nm (Thorlabs SFL1550) followed by and electronically controlled variable optical attenuator and a in-line polarization controller. In this way, we were able to finely control the intensity and the polarization of our LO, besides making the calibration procedure automatized. Before entering the heterodyne measurement, 10% of the LO is sent to a photodetector, for a continuous monitor of its intensity. In such way, any anomaly to the normal functioning of the LO can be noticed in real time, and deviations can be compensated during the post-processing. The optical heterodyne was realized with a commercial fiber integrated “90 degree hybrid”: one port is coupled to the LO while from the other is entering the vacuum state. The 90 degree hybrid mixes the signal with the LO and returns two pairs of outputs, featuring a *π*/2 phase shift. These optical signals, detected by a couple of high-bandwidth balanced detectors (1.6 GHz Thorlabs-PDB480C), are proportional to the quadratures of the signal, *q* and *p*. We sampled both signals coming from the detectors using a fast oscilloscope with 10 bits of resolution (LeCroy HDO 9404). The oscilloscope operated in burst mode, acquiring the analog signal at 10 GSps until the entire memory was completely filled. Then, the data are streamed to the computer via an Ethernet connection where it was post-processed. However, by using high resolution ADC and high throughput FPGA for real time processing, multi GBps real time extraction has been shown^34^.

## Electronic supplementary material

Supplementary Information

## Data Availability

The data that support the findings of this study are available from the corresponding author upon reasonable request.
