# Supplementary material for: Source-device-independent heterodyne-based quantum random number generator at 17 Gbps
Source: Nat Commun. 2018 Dec 18;9:5365. doi: 10.1038/s41467-018-07585-0 (PMC6299089; doi:10.1038/s41467-018-07585-0)
Supplement: Supplementary file 1 — Supplementary Information [file 41467_2018_7585_MOESM1_ESM.pdf]

Supplementary Information - Source-device-independent heterodyne-based quantum random number generator at 17 Gbps

Avesani et al.

SUPPLEMENTARY NOTE 1 - CALIBRATION

In the SDI framework we assume a trusted and characterized measurement device. In order to enforce that, before every run of the experiment we perform a calibration of our detection stage. This procedure is necessary for the evaluation of security, because it links the voltage output of the detectors to the relative quantities in the phase space, enabling us to calculate δ_q, δ_p.

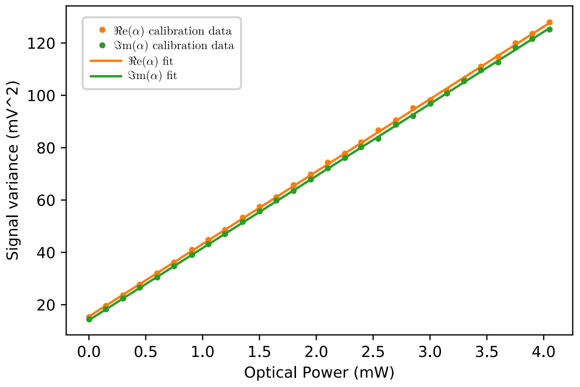

Supplementary Figure 1. The graph shows the linear dependence of the signal quadrature σ_V^2 as a function of the LO power.

The relation between the variance in physical units (Volts) and in shot-noise units is given by

$$\sigma_q^2 = \frac{\sigma_V^2}{kP_{\mathrm{LO}}} \tag{1}$$

where k is a constant to be determined. In the above equation σ_q^2 is the variance in shot-noise units, σ_V^2 is the variance in physical units and P_{LO} the power of the local oscillator. The constant k can be determined in the calibration phase: the vacuum is injected in the signal port of the heterodyne while the power of the LO, P_{LO}, is raised from 0 mW to the working power. During this process we record the variance of the electronic signal for each quadrature $\sigma_{V_q}^2$ and $\sigma_{V_p}^2$. From a linear fit we have:

$$\sigma_{V_{q,p}}^2 = m_{q,p}P_{\mathrm{LO}} + c_{q,p} \tag{2}$$

In an ideal condition (no electronic noise) the constants $c_{q,p}$ should be 0, however in any real experiment they don't vanish. In our convention, the theoretical quadrature variances in shot-noise units for the vacuum are given $\sigma_{q,p}^2 = \frac{1}{2}$, the constant k is obtained as $k_{q,p} = 2m_{q,p}$ Since we are not including the $c_{q,p}$ in the conversion factor $k_{q,p}$, we are considering the most conservative scenario, in which all classical noise is not trusted. Indeed, for a vacuum input state $|0\rangle$ and a given value of P_{LO}, the measured variances in shot-noise units are then given by

$$\sigma_{q,p}^2 = \frac{\sigma_V^2}{kP_{\mathrm{LO}}} = \frac{m_{q,p}P_{\mathrm{LO}} + c_{q,p}}{2m_{q,p}P_{\mathrm{LO}}} = \frac{1}{2} + \frac{c_{q,p}}{2m_{q,p}P_{\mathrm{LO}}} \tag{3}$$

which are always larger than $\frac{1}{2}$ for non-vanishing $c_{q,p}$. In this way the electronic noise (related to $c_{q,p}$) is regarded as noise on the source: it leads to an increase of the variances $\sigma_{q,p}$, thus lowering the min-entropy. Figure 3 of the main text clearly shows this effect: the reconstructed Q function is larger than the one expected for the vacuum because of this noise.

The calibration is performed automatically by the software that controls the QRNG: by varying the Variable Optical Attenuator (VOA), the power of the LO is changed from 0.01mW to 4.05mW, when measured with the monitor photodiode. For each power, the signal of the balanced detector is recorded and the variance σ_V^2 is estimated. As we can see in Supplementary Figure 1 the relation is linear for all the tested powers (i.e. we never reached the saturation of the detector's amplifiers). From the fit, $m_1 = (2.783 \pm 0.005) \cdot 10^{-2}\mathrm{V}^2/\mathrm{W}$ and $q_1 = (1.526 \pm 0.005) \cdot 10^{-5}\mathrm{V}^2$ for the slope and intercept of the first detector and $m_2 = (2.748 \pm 0.004) \cdot 10^{-2}\mathrm{V}^2/\mathrm{W}$ and $q_2 = (1.419 \pm 0.004) \cdot 10^{-5}\mathrm{V}^2$ for the second one. The errors are propagated in the estimation of the $H_{\min}(X|E)$ and the most conservative value in the 1 standard deviation confidence interval is used as a lower bound. Using a a more conservative 3 standard deviation confidence interval, the bound on the min entropy is reduced from 13.949 to 13.930 bits, for an equivalent secure generation rate of 17.41 Gbps.

SUPPLEMENTARY NOTE 2 - FILTERING, NOISE AND AUTOCORRELATION

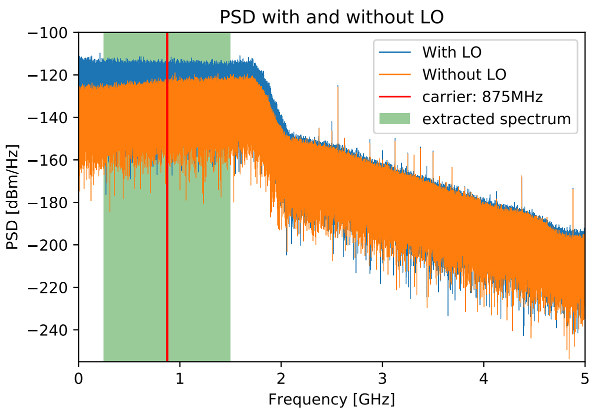

Supplementary Figure 2. Spectrum obtained from the detectors with or without the LO active. In green is highlighted the portion kept after the digital filtering and used for the generation. The peaks present after the 3dB point of the detectors are introduced by the oscilloscope at harmonics of the sampling frequency and are not present if the spectrum is obtained with an analog spectrum analyzer (HP 8561B).

To further reduce the classical noise from the detectors (at the expense of a reduced generation rate) we perform a filtering of the signal.

Supplementary Figure 2 shows the power spectral density of the signal produced by the detectors when the LO is turned on and when the LO is off. Although, the response seems uniform along the entire bandwidth of the detectors (1.6GHz), the initial part of the spectrum ($DC - 1$MHz) is affected by technical noise. In order to filter out this noise and enhance the signal-to-noise ratio, we have considered for the random generation only a window large 1.25GHz centered around 875MHz. With this selection, the gap is never lower than 9.6dB. The selection has been done digitally.

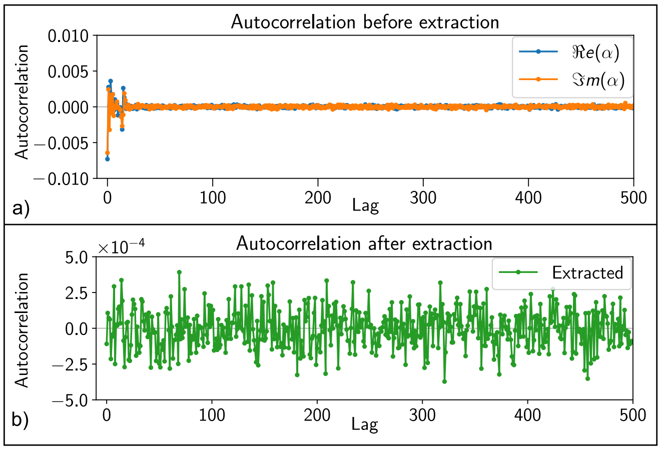

Supplementary Figure 3. Panel a) shows the autocorrelation measured for a sample of $5 \cdot 10^7$ raw numbers before the extraction while panel b) shows the autocorrelation of the extracted numbers. The spikes present in the first lags before the extraction are due to the noise introduced by our sampling equipment. However, they are completely absent after the extraction.

However, employing a Brick-wall filter in the frequency domain, inevitably induces correlation in the time-domain of our signal: indeed we observe a "sinc" dependence in the autocorrelation, as expected from the Wiener-Khinchin theorem. The correlation is removed by undersampling the signal in such a way to match the first zero of the autocorrelation function. Supplementary Figure 3 shows the residual autocorrelation after the downsampling, before and after the randomness extraction for a run of $5 \cdot 10^7$ samples. The results, even before the extraction, are good, with values always below $7.5 \cdot 10^{-3}$ and typically below $1 \cdot 10^{-4}$, except for the first lags. The value of the first lag is due to noise introduced by the oscilloscope at harmonics of its sampling rate frequency.

In Supplementary Figure 2, these distortions are clearly visible at high frequencies, where there is no contribution from the signal. However, after the extractor, all the classical noise is eliminated and the autocorrelation is completely flat, also for the initial lags.

SUPPLEMENTARY NOTE 3 - DIGITALIZATION AND RANDOMNESS EXTRACTION

As described in the main text, the analog electric signals coming from the detector are digitalized by an high bandwidth and high resolution oscilloscope, in order to be further post-processed. The oscilloscope doesn't work in real-time but in burst mode, meaning that the signals are sampled at 10 GSps until the entire memory is completely filled. Then, the data are streamed to the computer via an Ethernet connection. Then, the filters discussed in Section are applied off-line. Finally, always offline, the randomness extraction is applied to the filtered data. We implemented the fast computable two-universal hash function introduced in [1], then we used it to extract the final numbers from the filtered samples. We calibrated the extractor with the value obtained bounding $H_{\min}(X|\mathcal{E}$ and then we extracted, using a PC, $\approx 5.18 \cdot 10^{10}$ random numbers from an initial set of $7.5 \cdot 10^{10}$ raw numbers. As we pointed out in the main text, the finite size effects and the block size are not relevant for the protocol, since the conditional quantum min-entropy $H_{\min}(X|\mathcal{E})$ characterizes the one-shot private randomness that can be extracted.

SUPPLEMENTARY NOTE 4 - STATISTICAL TESTS

In order to check for problems in our implementation we performed some statistical test on the generated numbers. We tested them with the NIST [2] and "dieharder" suite [3]: in both cases all the tests were passed, as we can see in Supplementary Table I,II. Passing these tests doesn't certify the randomness, but only shows that some patterns are not present in the analyzed data. However, since our QRNG is supposed to pass all of them, is a way to double-check that our setup is working as expected.

Test's name	P-value	Result
diehard birthdays	0.398	PASSED
diehard operm5	0.391	PASSED
diehard rank 32x32	0.414	PASSED
diehard rank 6x8	0.767	PASSED
diehard bitstream	0.529	PASSED
diehard opso	0.655	PASSED
diehard oqso	0.758	PASSED
diehard dna	0.731	PASSED
diehard count 1s str	0.482	PASSED
diehard count 1s byt	0.361	PASSED
diehard parking lot	0.515	PASSED
diehard 2dsphere	0.484	PASSED
diehard 3dsphere	0.739	PASSED
diehard squeeze	0.580	PASSED
diehard sums	0.140	PASSED
diehard runs	0.478	PASSED
diehard runs	0.316	PASSED
diehard craps	0.348	PASSED
diehard craps	0.937	PASSED
marsaglia tsang gcd	0.504	PASSED
marsaglia tsang gcd	0.444	PASSED
sts monobit	0.204	PASSED
sts runs	0.716	PASSED
sts serial	0.151	PASSED
rgb bitdist	0.056	PASSED
rgb minimum distance	0.043	PASSED
rgb permutations	0.068	PASSED
rgb lagged sum	0.019	PASSED

Supplementary Table I. Result of Dieharder test suite on the extracted random numbers. In the case of multiple tests in a category, the smallest have been reported.

Test's name	P-value	Result
Frequency	0.980	PASSED
BlockFrequency	0.323	PASSED
CumulativeSums	0.819	PASSED
CumulativeSums	0.265	PASSED
Runs	0.187	PASSED
LongestRun	0.864	PASSED
Rank	0.372	PASSED
DFT	0.341	PASSED
NonOverlappingTemplate	0.016	PASSED
OverlappingTemplate	0.748	PASSED
Universal	0.381	PASSED
ApproximateEntropy	0.509	PASSED
RandomExcursions	0.315	PASSED
RandomExcursionsVariant	0.047	PASSED
Serial	0.318	PASSED
LinearComplexity	0.373	PASSED

Supplementary Table II. Result of NIST test suite on the extracted random numbers. In the case of multiple tests in a category, the smallest have been reported.

SUPPLEMENTARY REFERENCES

[1] Frauchiger, D., Renner, R. & Troyer, M. True randomness from realistic quantum devices Preprint at http://arxiv.org/abs/1311.4547 (2013).

[2] Bassham III, L. E. *et al.* Sp 800-22 rev. 1a. A statistical test suite for random and pseudorandom number generators for cryptographic applications. (2010). URL https://www.nist.gov/publications/statistical-test-suite-random-and-pseudorandom-number-generators-cryptographic.

[3] Brown, R. G., Eddelbuettel, D. & Bauer, D. Dieharder: A Random Number Test Suite (2013). URL https://webhome.phy.duke.edu/~rgb/General/dieharder.php.
